# Implementation evaluation of a collective impact initiative to promote adolescent health in Oklahoma County, USA

**DOI:** 10.1186/s12889-021-12482-1

**Published:** 2022-01-10

**Authors:** Whitney R. Garney, Sonya Panjwani, Kelly Wilson, Kristen E. Garcia, Sharayah Fore, Shelby C. Lautner, Laura Lang, Brittney Criswell, Ronneal Mathews

**Affiliations:** 1 Department of Health and Kinesiology, Texas A&M University, 4243 TAMU, College Station, TX 77843 USA; 2 Thrive: Sexual Collective for Youth, 3000 United Founders Blvd #247, Oklahoma City, OK 73112 USA

**Keywords:** Adolescence, Collaboration, Community capacity building, Process evaluation, Teenage pregnancy prevention

## Abstract

**Background:**

The teenage birth rate in the USA has considerably decreased in recent decades; however, more innovative, collaborative approaches are needed to promote adolescent health and prevent teenage pregnancy at the community level. Despite literature on the promising results of the collective impact (CI) model for health promotion, there is limited literature on the model’s ability to reduce teenage pregnancies in a community. The Central Oklahoma Teen Pregnancy Prevention Collaboration is applying the CI model to foster collaboration among multiple stakeholders with the goal of increasing community and organizational capacity to improve adolescent health outcomes. This paper reports the findings from the initiative’s implementation evaluation, which sought to understand whether the CI model improved collaboration among organizations and understand barriers and facilitators that affected program delivery.

**Methods:**

Program implementers and evaluators jointly developed research questions to guide the intervention and evaluation design. The Consolidated Framework for Implementation Research (CFIR) was used to assess program components including the intervention characteristics, organization setting, community setting, facilitator characteristics, and the process of implementation. Primary sources of data included performance measures, meeting observations (*n* = 11), and semi-structured interviews (*n* = 10). The data was thematically analyzed using CFIR constructs, community capacity domains, and the five constructs of CI.

**Results:**

Key findings include the need for shortened meeting times for meaningful engagement, opportunities for organizations to take on more active roles in the Collaboration, and enhanced community context expertise (i.e., those with lived experience) in all Collaboration initiatives. We identified additional elements to the core constructs of CI that are necessary for successful implementation: distinct role identification for partner organizations and incorporation of equity and inclusivity into collaboration processes and procedures.

**Conclusions:**

Results from this implementation evaluation provide valuable insights into implementation fidelity, participant experience, and implementation reach of an innovative, systems-level program. Findings demonstrate the context and requirements needed to successfully implement this innovative program approach and CI overall. Additional core elements for CI are identified and contribute to the growing body of literature on successful CI initiatives.

**Supplementary Information:**

The online version contains supplementary material available at 10.1186/s12889-021-12482-1.

## Background

While teenage birth rates reached a record low in the United States of America (USA) in 2017 (18.8 per 1000 women), data suggests disparities exist among racial/ethnic groups and in certain geographic locations [1]. For example, based on data from 2017, Hispanic teenage girls and non-Hispanic Black teenage girls were more than twice as likely to become pregnant when compared to their non-Hispanic White counterparts [2]. One study found that the greatest disparity between Black and White teenage pregnancy rates existed in the early adolescent years and then decreased by nearly 50% during later adolescence [3]. Numerous negative social outcomes for those that became pregnant earlier in adolescence were more pronounced, including lower educational attainment and increased risk of substance misuse, depression, and homelessness [4–6].

The challenges related to teenage pregnancy are ecological and complex, with no single solution to solving them. There is a network of interrelated influencing factors that give rise to complex collective behaviors, including those related to an individual’s sexual risk [7]. Furthermore, the resulting behaviors are not stagnant; they are constantly evolving and changing over time [8, 9]. Contributing components include individual-level factors such as socioeconomic status and sexual health knowledge; interpersonal factors such as a family history of teenage pregnancy and social support; community-level factors including geographic location, access to community resources, and systemic inequalities that result in racial/ethnic disparities, among others [10]. Therefore, preventive efforts must consider the issue at the macro-level by targeting multiple ecological levels [7, 10].

When viewing adolescent health and teenage pregnancy from a systems perspective, we seek to understand the interactions and impacts that unintended pregnancy has on both teenage parents and their children. One example is the reinforcing feedback loop between socioeconomic status and teenage pregnancy. Becoming a teenage parent is a significant contributor to high school dropout rates among women, with only ~ 50% of teenage mothers receiving a high school diploma before 22 years of age [11]. Without quality education, teenage mothers can experience economic hardships [2]. Research also shows that teenagers of low socioeconomic status are more likely to become pregnant during their high school years [12]. Therefore, just as teenage pregnancy immediately affects the mother-child dyad, it also has long-term consequences because the children of teenage parents are at a higher risk of experiencing an unplanned pregnancy [12].

Public health researchers and practitioners are working to advance efforts, interventions, and policies aimed at promoting adolescent health and preventing teenage pregnancy. However, many of these efforts use individual-level theories to target individual and interpersonal risk factors immediately affecting youth [13]. Currently, the field lacks systems-level approaches that seek to understand and modify both individual behaviors, as well as the interactions between individual behaviors and the environmental context in which they occur [13]. Systems-level techniques that addresses both teenage pregnancy risk factors and promote protective factors is critically needed to implement effective preventive and health promoting efforts [2, 10].

### Community context

Geographically, the state of Oklahoma is disparately impacted by teenage pregnancy. It has the fifth highest teenage birth rate in the nation, with Oklahoma County ranking highest in the number of teenage births in the state [14]. Due to concentrated funding and efforts among committed organizations, the teenage birth rate in Oklahoma County has decreased by 42% from 2013 to 2018 [1]. Despite this progress, the teenage birth rate in Oklahoma County (29.8 per 1000 females) is still higher than the rate in the state overall (27.1 per 1000 females) and nationally (18.8 per 1000 females) [1].

This complex issue has significantly affected Oklahoma socially and economically. It is estimated that $29.2 million in annual savings would result from avoiding the 3504 teenage births that occurred in Oklahoma in 2019 [14]. This estimation takes into account public savings resulting from reduced Medicaid spending related to prenatal care, labor, delivery, postpartum care, and a year of infant care as well as public assistance such as Temporary Assistance for Needy Families, the Special Supplemental Nutrition Program for Women, Infants, and Children, and the Supplemental Nutrition Assistance Program during pregnancy and the year following [14]. The social impacts of teenage pregnancy include educational setbacks, lower income attainment, and a decrease in social capital. Economically, this translates to less taxes paid locally and poorer labor-market outcomes [15]. Additional targeted efforts are needed to make further improvements in the teenage birth rate and promote optimal health for youth [10, 16]. These efforts have the potential to not only increase the community’s ability to effectively utilize its resources, but such efforts will also address environmental and protective factors that could greatly decrease the teenage birth rate in these communities [10].

### The collective impact model

Collective impact (CI) is an effective strategy for addressing complex systems-level issues [8, 9, 17, 18]. CI builds on previous interorganizational collaboration frameworks and has been used to achieve large-scale change and systems-level impact [9, 17]. Unique from other community-based partnership models, CI is based on five key constructs that drive its activities. These constructs include a common agenda, shared measurement systems, mutually reinforcing activities, continuous communication, and a central infrastructure or backbone organization(s) [8, 9]. A definition for each of these constructs is provided in Table 1. These constructs combine to create an overall CI model that brings together various stakeholders for more than just a collaboration; it helps them perform at a higher level and increase their capacity to address social change [18].

**Table 1:** The Central Oklahoma Teen Pregnancy Prevention Collaboration’s Definition for each of the Collective Impact Constructs (Kania and Kramer, [8])

Collective impact constructs as intervention activities (question 4)	Collective impact construct definition	Central Oklahoma Teen Pregnancy Prevention Collaboration definition including who will deliver the component (question 6), modes of delivery (question 7), when and how much will be delivered (question 8), and tailoring of the component (question 9)
Common Agenda	Agreement from all member organizations on the primary goals of the collaborative.	Reduce the teen birth rate in Oklahoma County by one-third by 2020
Shared Measurement	Ensure that data is systematically collected and reported based on a set of indicators that can be used by all members of the collaborative to uniformly assess programs and encourage accountability.	Shared data measures developed by Collaboration members for education, medical, and community settings that monitors the increase in access to information about preventing pregnancy, access to and usage of contraception by sexually active youth and young adults, and strengthens protective factors for youth at risk of teen pregnancy within the community
Mutually Reinforcing Activities	Acknowledges that though different partners have distinct roles in the system, their activities are linked to an overarching goal that is collaboratively determined.	Partner activities in education, medical, and community settings that encompassed sexual and reproductive health education in schools, increased access to medical services, and youth development programs in the community
Continuous Communication	Involves regular interactions that build relationships, trust, and a shared vocabulary.	Open channels of communication to ensure coordination of activities, highlighting successes of the Collaboration to the community, and receive feedback on Collaboration functioning
Backbone Support	Establishes a dedicated staff, separate from collaborative partners, that coordinates, facilitates, supports, guides, and mediates the collaborative effort.	Purpose of Thrive: guide vision and strategy, build a movement in the community, support aligned activities, establish shared measurement practices, advance policy, mobilize funding

One of the most cited CI initiatives took place in Somerville, MA. This initiative, known as *Shape Up Somerville*, was born out of a community-based participatory project focused on obesity prevention. This effort targeted the availability of healthy food and opportunities for physical activity in children’s school environments [19]. Organizational partners came together to align individual activities and create a community movement to address this issue. Programmatic strategies ranged from the individual-level to the systemic or community-level, including afterschool cooking lessons, negotiations for enhanced school lunches, and a city walkability and bikeability ordinance [19]. The initiative became a model for systems-level interventions promoting health and wellbeing due to the magnitude of the positive change in childhood obesity rates in the intervention community compared to control communities [18].

### Central Oklahoma Teen Pregnancy Prevention Collaboration

Oklahoma County’s teenage pregnancy prevention efforts began well over a decade ago, driven by philanthropic organizations wanting to create positive social change in their community. In 2006, five organizations began working together to provide educational programs and clinical services for teenagers in the areas of Oklahoma County where teenage births were the highest. In 2013, the group formed the Central Oklahoma Teen Pregnancy Prevention Collaboration (the “Collaboration”). These organizations included both public and private entities such as the county health department, nonprofits, and federally qualified health centers. To accomplish their strategic goal of reducing the teenage birth rate by one-third, in 2019, the Collaboration adopted the CI model as an innovative approach that would allow individuals and organizations from different sectors in the community to work together effectively. Guided by the model, key leaders then established the organization, Thrive, to serve as the backbone organization.

The Collaboration’s initial structure focused on building community capacity through incorporating three distinct sectors or “pillars” to guide its activities and take collective action [20]. These three focus areas formed the basis of working groups comprised of medical, education, and community sector professionals and community members from diverse organizations and backgrounds, all of whom had a passion for adolescent health promotion and teenage pregnancy prevention work. This shared mission brought these organizations and individuals together and increased the magnitude of their impact through their collective efforts [21].

Each working group pursued its own objectives. For example, the medical working group worked to identify and remove access barriers to medical services for teenagers, create referral networks between trusted providers, and ensure that the information distributed from the Collaboration was medically accurate. The education working group focused on ensuring delivery of medically accurate, age-appropriate, trauma-informed, user-centered information in schools and the community. The community working group focused on monitoring and sharing community needs while also authentically integrating community members with lived experiences into the Collaboration’s structure. Although the focuses of the working groups were different, their frequent, supportive interactions allowed for cooperative decision-making as the Collaboration worked towards its collective goals. A modified version of the Template for Intervention Description and Replication (TIDieR) framework for describing interventions [see Additional file 1] was used to illustrate how the Collaboration defined the five constructs of CI and to further describe the structure and focus of the Collaboration (see Table 1) [8, 22]. The TIDieR framework questions are relevant to describing each of the key components are illustrated within the table.

### Study framework and aim

This study presents the results from the Collaboration’s implementation evaluation, which was conducted to evaluate how effective the Collaboration’s current practices were, how it had and will continue to contribute to the promotion of optimal health for teenagers, and to adjust the CI framework to become more effective in Oklahoma County. Using performance measure data supplemented by qualitative data sources, the results presented here explored whether the CI model had improved collaboration among partner organizations and identified the barriers and/or facilitators impacting program delivery. This comprehensive evaluation aims to provide useful information to similar preventive, community-based initiatives interested in making systems-level changes.

## Methods

This implementation evaluation was designed to answer two evaluation questions:

- *Evaluation Question 1:* To what extent does the CI model improve collaboration for adolescent health promotion and teenage pregnancy prevention in Oklahoma County?
- *Evaluation Question 2:* What were the barriers that influenced the implementation of the CI model for the Collaboration?

### Implementation evaluation design

To assess short-term outcomes and the overall program structure, an implementation evaluation was jointly developed by a team of external evaluators and program implementers [23]. The evaluation team used a comprehensive, systematic, six-step, iterative process to determine whether the intervention was implemented as planned and capable of furthering its goal of increasing community capacity to prevent teenage pregnancies in Oklahoma County [23]. To assess the implementation process of the Central Oklahoma Teen Pregnancy Prevention Collaboration’s intervention, evaluators used three primary sources of data: 1) adapted performance measures required by the funder, 2) meeting observations for program fidelity, and 3) semi-structured interviews with key Collaboration partners [24]. A descriptive summary of each data collection tool, the evaluation question it corresponds to, and the theoretical basis for the tool is shown in Table 2. The Texas A&M University Institutional Review Board approved the protocol and materials prior to data collection. All methods were performed in accordance with the relevant guidelines and regulations.

**Table 2:** Data Collection Tools

Data Collection Tool	Evaluation Question	Description	Theoretical Framework
Performance Measures	Evaluation Question 1 Evaluation Question 2	Assessed Collaboration’s activities (e.g., meetings, trainings)	N/A
Meeting Observations	Evaluation Question 1	Evaluated the effectiveness of Collaboration meetings, including leadership, participation, decision making, conflict resolution, productivity, and data sharing	Goodman’s (1996) Meeting Effectiveness Inventory Collective Impact Constructs
Semi-Structured Interviews	Evaluation Question 1 Evaluation Question 2	Open ended interview protocol to assess the structure of the Collaboration, contextual community factors that may affect the Collaboration’s ability to make a change in their community, and the implementation process	Collective Impact Core Constructs Consolidated Framework for Implementation Research Community Capacity Domains

### Data sources

#### Performance measures

The funder-required performance measures were adapted by evaluators and Collaboration leaders for relevance to a systems-level intervention and used as a progress metric. The program-specific measures focused on the Collaboration’s activities and its implementation of community-wide initiatives. The performance measures served as a way to measure the systems-level CI implementation process and were collected during three reporting periods (T1: April 1, 2019-September 30, 2019; T2: October 1, 2019-March 31, 2020; T3: April 1, 2020-September 30, 2020). Additional file 2 contains the breakdown of the adapted performance measures relevant to this study.

#### Observation data

##### Observation tool

To measure the fidelity and quality of implementation and evaluate the effectiveness of Collaboration meetings, evaluators undertook a four-step process for fidelity measurement development [24]. First, relevant components for monitoring were identified [24]. Evaluators used Goodman’s Meeting Effectiveness Inventory [25] as a validated tool that would provide the basis for observations. The original tool is used to rate meetings for leadership, participation, decision making, conflict resolution, and productivity [25]. Existing questions were cross-referenced with the five CI constructs, and additional questions were added to assess constructs that were not addressed [see Additional file 3] [8, 25]. Second, evaluators and program implementers met to identify a member of the external evaluation team that had no involvement in the planning or facilitation of meetings who would serve as the external observer [24]. The external observer then used the modified tool throughout T2 and T3 to observe 11 Collaboration meetings (12%) and to rate the quality of implementation of the CI framework. These included both individual working group meetings and overall collaboration meetings. Both quantitative and qualitative data were obtained from the tool, with some responses to questions requiring election based on a Likert-scale of 1 (poor) to 5 (excellent), and others requiring open-ended responses for a more in-depth assessment. An overall aggregate score ranging from 1 (poor) to 5 (excellent) was calculated from the average score of each of the scaled questions. Finally, a summary score and report for the ratings were developed.

##### Data analysis

A member of the evaluation team analyzed meeting observation data to provide feedback to program implementers on a continuous basis (i.e., after each meeting) and understand how well meetings adhered to the five constructs of CI. The evaluator categorized the meetings based on type, analyzed the individual questions listed on the observation form, and calculated an average score across all observations for each question. Inductive thematic analysis was used to analyze qualitative responses and provide insight into the strengths and weaknesses of meeting facilitation [26].

#### Semi-structured interviews

Evaluators conducted interviews to obtain feedback on Collaboration functioning, implementation of initiatives, and perceived programmatic impact from key Collaboration members. Potential interviewees were identified by Collaboration leadership based on number of years of Collaboration involvement and level of engagement with Collaboration activities. These organizations participated at a higher level (i.e., attended the majority of meetings, implemented and participated in community events) and were chosen based on their knowledge of the Collaboration’s history and future directions.

In mid-October 2019, 22 potential interviewees across 13 organizations were invited by a member of the evaluation team to participate in an interview and schedule a time to meet via an online scheduling link. In the initial recruitment email, potential interviewees were provided with information about the purpose of the interviews and other important details through an information sheet and consent form. If individuals did not register for an interview upon first contact, evaluators sent a reminder email. A total of 10 interviews (*n* = 10) were conducted with Collaboration members representing healthcare (50%), nonprofit (30%), and government (20%) organizations, resulting in a response rate of 45%.

##### Interview tool

Evaluators developed an open-ended interview script based on published CI core constructs, community capacity domains, and the Consolidated Framework for Implementation Research (CFIR) [8, 20, 27]. CI constructs were used to assess the structure of the Collaboration, community capacity was assessed to gain information about community readiness and contextual factors that may affect change, and the CFIR was used to assess the implementation process. Additional file 4 includes the interview questions cross-referenced with the construct assessed.

##### Interview protocol

Interviews were conducted over the telephone by either one of two members of the evaluation team who had masters-level training in public health. Interviewers reviewed the scripts and completed two mock interviews prior to conducting interviews to rehearse the interview administration process and determine whether the questions would elicit the intended information [28]. Additionally, collaboration leaders reviewed interview scripts for relevance and understandability. Each interview lasted between 30 and 60 min. Participants knew the general premise of the interviews beforehand; however, questions were not provided to the interviewees before the calls. Participants returned signed consent forms prior to the start of the interviews, and at the beginning of each call, the interviewer asked for participant consent to be recorded. Upon consent, interviews were audio-recorded and transcribed by an external transcription firm with a signed confidentiality agreement. A note-taking template was available in the event a participant declined to be recorded. No interviewees declined to be recorded.

##### Data analysis

In December 2019, evaluators conducted a thematic analysis of interview data using an open coding scheme to identify emergent codes and overall themes [29]. The qualitative analysis was conducted by two trained members of the evaluation team, in which both evaluators reviewed the transcripts independently to identify key points and then came together to compare data [30, 31]. Discrepancies were resolved through consensus. The evaluators developed themes from the coded data for each question independently, and then for the overall dataset across questions. Themes were then viewed through the lens of CFIR, community capacity domains, and the five CI constructs to achieve further context and understanding.

### Data synthesis

Data was synthesized from data collection tools to answer each evaluation question. Performance measure domains and key themes from interviews were used to identify categories for this analysis. Evaluators began by reviewing performance measures and selecting those related to the evaluation questions. Then, a summary table was designed containing relevant domains from the performance measures and key themes from semi-structured interviews as the basis for analysis. Categories were placed in columns and notes from all data sources that corresponded to these categories were placed within each row. Interview transcripts were reviewed to extract quotes that provided support for each of the domains.

## Results

Both qualitative and quantitative data demonstrated the extent to which the CI model improved collaboration for adolescent health promotion and teenage pregnancy prevention in Oklahoma County. Evaluators used adapted performance measures to determine the change in dosage, engagement and training, and fidelity and implementation quality from April 1, 2019 to September 30, 2020; see Table 3. Semi-structured interviews (*n* = 10) provided an in-depth understanding of how the Collaboration engaged the community in its activities, the partners’ experiences with the CI model, and key areas of improvement.

**Table 3:** Performance Measure Data

Performance Measure	T1 (April 1, 2019-September 30, 2019)	T2 (October 1, 2019-March 31, 2020)	T3 (April 1, 2020-September 30, 2020)
**Dosage**			
# of Organizations that Attended 75% of Meetings	70 Organizations	71 Organizations	30 Organizations
Average Organization Meeting Attendance	73.8%	91.9%	93.6%
Average Length of Collaboration and Working Group Meetings	146 Minutes	101 Minutes	68 Minutes
Number of Meetings and Working Groups Implemented per 6 Month Period	24 Meetings	54 Meetings	39 Meetings
**Engagement and Training**			
Number of Core Organizations Invited to be Engaged	22 Organizations	23 Organizations	23 Organizations
Number of Organizations Engaged During Reporting Period	10 Organizations	14 Organizations	16 Organizations
Number of Organizations Trained Through Engagement with the Collaboration	4 Organizations	8 Organizations	4 Organizations
Number of Trainings Conducted by and for the Collaboration	9 Trainings	10 Trainings	5 Trainings
**Fidelity and Quality**			
Overall Quality of Programming	–	5	4.66
Number of Meetings or Working Groups Observed	–	1 Meeting	10 Meetings
Number of Meetings Planned for Each Working Group	24 Meetings	16 Meetings	21 Meetings
Number of Meetings Conducted for Each Working Group	24 Meetings	14 Meetings	21 Meetings
Items Implemented Through Working Group During Reporting Period	1 Item	4 Items	3 Items

### Dosage

The number of organizations that attended at least 75% of meetings or activities to which they were invited remained almost the same for T1 and T2 (70 organizations and 71 organizations, respectively), but decreased by more than half during T3 (30 organizations). Qualitative data revealed that the decrease can be attributed to Thrive being sensitive to its partners’ ability to participate due to COVID-19, as well as Thrive recognizing challenges with relationship building and meeting facilitation in a new, virtual environment. Thus, fewer organizations were invited to meetings to accommodate partner organizations’ changing priorities. Average meeting attendance increased from T1 to T3 (T1: 73.8%, T2: 91.9%, T3: 93.6%). The average length of Collaboration and working group meetings decreased over time (T1: 146 min, T2: 101 min, T3: 68 min), which was attributed to better meeting facilitation and the transition to virtual meetings due to the COVID-19 pandemic. The overall number of meetings increased from T1 to T2 (24 meetings to 54 meetings) followed by the average number of meetings for these two time points held during T3 (39 meetings).

### Engagement and training

A key set of core organizations were engaged and trained in individual-level teenage pregnancy prevention programs, sexual health education strategies, CI, and continuous communication and relationship building to establish mutually reinforcing activities across multiple sectors. Engagement in this context is defined as those organizations that had high meeting attendance and/or were involved in a specific project during the reporting period. Across all time points, approximately the same number of core organizations were invited to be more actively engaged in the Collaboration’s activities; however, six more organizations were actively engaged in T3 than in T1. Nearly the same number of trainings were conducted during the first two reporting periods (T1: 9 trainings, T2: 10 trainings), but half as many trainings were held during the third reporting period (5 trainings). Conversely, twice as many organizations were trained in T2 than in T1 and T3. One interviewee identified the training component as one of the greatest strengths of the Collaboration. They stated, *“I would guess [the] biggest success would be they did a Teen Speak training and they train community members. And they’ve done one Teen Speak training for parents and caregivers.”*

### Fidelity and quality

Performance measure data related to meeting implementation and effectiveness was used to determine implementation fidelity and quality. Meetings were held with the entire Collaboration as well as the individual working groups (medical, community, and education). During T1, the number of planned and conducted working group meetings remained the same (24); however, during T2, the number of planned working group meetings decreased to 16, with 14 meetings being held. Two meeting cancellations occurred at the onset of the COVID-19 pandemic. In T3, the number of meetings planned and conducted increased to 21 meetings. Meeting assessment data revealed that the greatest strength of meetings was attendee cohesiveness that allowed for open communication and ease of working together, with the area of greatest improvement needed being the clear identification of meeting goals in the meeting agenda. The benefit and challenges of working groups were demonstrated by one of the co-chairs of the Community Working Group:

*“[The strategic plan] gave us a framework of things that we all agreed were important to be working on.* […] *So, having those goals set with some action items that we all wanted to work on really did give some guidance and direction and purpose to these working groups. But I will tell you, there were some frustrations with some of the activities we had all agreed to because some of them were just harder to figure out how to get them off the ground than others.”*

### Community engagement

Recently, there has been a movement toward increasing community engagement within the CI model. This removes barriers between community members and leadership and results in more effective implementation [32]. Qualitative interviews provided insight into the extent to which community members were aware of the initiative and how well the Collaboration engaged a broad base of community members and organizations. This feature aligns with the inner and outer settings of the CFIR constructs and addresses two key dimensions of community capacity building, building a sense of community and interorganizational networks [20, 27]. A majority of interview respondents felt that the community had some knowledge about the Collaboration; however, this awareness was limited to the education, healthcare, nonprofit, medical, and government sectors. Individuals emphasized in their interviews that the Collaboration was in the process of expanding engagement through outreach to other sectors using community events and newsletters to broaden the types of organizations involved, as well as involving context experts in all aspects of the Collaboration. For example, one respondent stated, *“[The Collaboration] reaches out to different community agencies. They make sure they have someone representing different aspects of people’s health, not just access to birth control, but access to education, and access to resources for homeless teenagers. They make sure they cover the breadth of the needs.”* Others described the Collaboration’s engagement plan as reaching the “heavy hitters” within the community including context experts and incorporating new members in leadership roles.

### Partner experiences

Interviewees were also asked about their experience with the CI model including the successes and challenges faced by the Collaboration. All interviewees identified the greatest success of the Collaboration as its contribution to the reduction of the Oklahoma County teenage birth rate by one-third. They also felt that the Collaboration had successfully leveraged its ability to bring people together and connect them to resources. One respondent shared that they *“think [the Collaboration] is a great example and probably one of only a few in Central Oklahoma where the model of collective impact is actually working.* […] *This one is showing true progress where others are not.”* Conversely, respondents identified that one of the greatest challenges of the Collaboration involved the difficulty in establishing a joint approach, a lack of communication designed to give people an equal voice, and role clarification. As described by one interviewee, *“With this many organizations working together, some things will get lost in translation, lost in the ability for us to actively communicate with each other. And that can be frustrating.* […] *It can be difficult for everyone to get on the same level.”*

## Discussion

The purpose of this evaluation was to understand the implementation of a CI initiative aimed at improving collaboration between organizations working to enhance adolescent health outcomes and prevent teenage pregnancies in Oklahoma County, USA. The results from this evaluation add to the literature on CI evaluation, identify priorities for emerging CI initiatives, and shed light on ways this model can be applied and sustained. Furthermore, this evaluation supports the implementation of community-level programs and can be used by other interorganizational collaborations interested in making systems-level changes within their community.

### Implications of the use of collective impact to address a public health issue

The CI framework provided a foundation to enhance interorganizational collaboration across partners in the Central Oklahoma Teen Pregnancy Prevention Collaboration. Decreased meeting lengths, increased opportunities to meaningfully engage in the Collaboration (e.g., through events or leadership roles), connecting organizations to resources, and increased community awareness and involvement in the Collaboration all proved to be useful for building a community movement to address teenage pregnancy. By decreasing meeting length, both intentional discussion and partner cooperation were improved, and attendee time and ability to stay engaged were respected. Furthermore, community engagement emerged as a critical aspect of the initiative; this supports research showing that a key feature of CI is the involvement of community members to bridge the disconnect between initiatives led by content experts and those with lived experience (i.e., context experts) [32]. Finally, indicators of successful CI implementation are shown through the ability of a collaborative to integrate the efforts of organizations from multiple sectors and make population health improvements, both of which were demonstrated through the work of the Collaboration [33]. The combination of these key success factors has assisted the Collaboration to build momentum from its initial five founding organizations to the 70+ organizations engaged today [33].

The complexity of teenage pregnancy prevention requires a coordinated community response and reflects the importance of employing a model such as CI to successfully address the issue [8, 9, 18]. The CI model has been widely used across multiple areas, including education, poverty reduction, obesity, physical activity, among others, and has been shown to provide a useful framework for the integration of systems to promote optimal health [34–38]. This model is useful in that it focuses on principles of collaboration, coalition development, and community organization [9, 39]. Because the impact of teenage pregnancy is not isolated to affected teenagers, it is important to consider systems-level changes to address the potential ripple effects of this issue impacting the wider community. Not only does this system focus improve individual outcomes for teenagers, it also benefits the community overall through the reductions in the use of public services, improvement of educational outcomes, and the production of a more capable and available workforce that can contribute to the community.

### Organizational considerations for implementing collective impact

Data from this evaluation revealed key areas of improvement including communication, relationship building, and defining and expanding who has a seat at the table. These key themes were identified through partner feedback and continuous monitoring, which proved to be critical to identifying the structural and process improvements needed in the Collaboration. Steps taken to improve Collaboration policies and procedures included better communication, clarification of decision-making processes, and creating well-defined roles for Collaboration partners. Furthermore, process improvements helped in preventing activities from becoming siloed, an issue identified in the early stages of the Collaboration. Lastly, while not cited as a part of the key constructs of CI, embedding equity and inclusivity into the structure of the Collaboration was critical to providing all stakeholders with a voice in the initiative and allowing them the opportunity to contribute to the community teenage pregnancy prevention movement.

### Limitations

This implementation evaluation is not without limitations. As with all community-based initiatives, evaluators faced difficulties in scheduling interviews with partner organizations due to their active schedules. The Collaboration also had many activities going on during this time (e.g., strategic planning, other simultaneously occurring data collection activities) that could have affected the low response rate. Evaluators took this into account and extended the data collection window to accommodate for partners’ schedules. Furthermore, observations were conducted by a single observer; this could lead to potential bias. However, evaluators attempted to account for this by using a validated tool and utilizing an observer from the external evaluation team that was not involved in the meeting planning and facilitation process. Additionally, this evaluation took place during the beginning stages of implementing the CI model for this Collaboration; for this reason, structures, processes, and procedures had not been fully defined and partner organizations’ scope of work narrowed and shifted throughout the evaluation. While this called for multiple pivots in terms of evaluation activities, it was important to capture these changes to continuously improve the work of the Collaboration. Finally, required performance measures were not released by the funder until July 2019, which required program implementers and evaluators to backtrack to calculate measures for the first time point. Performance measures also had to be adapted to better fit the systems-level intervention.

## Conclusions

This study provides important insights for future implementation of community-wide initiatives aimed at making population-level changes. Application of the key constructs of CI can provide a foundation to improve cross-sector collaboration and meaningfully engage community members in an innovative way to address complex health problems such as teenage pregnancy. However, as gleaned from the findings in this study, expanding these constructs to incorporate factors such as role identification and equity could prove useful. Future research to systematically identify these additional factors will expand the knowledge base of CI and improve implementation.

## Supplementary Information

**Additional file 1.** TIDieR Checklist

**Additional file 2.** Adapted Performance Measures for the Central Oklahoma Teen Pregnancy Prevention Collaboration.

**Additional file 3.** Adapted Meeting Effectiveness Assessment.

**Additional file 4.** Semi-Structured Interview Protocol.

## Data Availability

The datasets used and/or analyzed during the current study are available from the corresponding author on reasonable request.

## Abbreviations

CI: Collective impact
CFIR: Consolidated Framework for Implementation Research
TIDieR: Template for Intervention Description and Replication
USA: United States of America
