# Supplementary material for: Implementation evaluation of a collective impact initiative to promote adolescent health in Oklahoma County, USA
Source: BMC Public Health. 2022 Jan 10;22:57. doi: 10.1186/s12889-021-12482-1 (PMC8743353; doi:10.1186/s12889-021-12482-1)
Supplement: Supplementary file 1 — Additional file 1. TIDieR Checklist [file 12889_2021_12482_MOESM1_ESM.docx]

**Additional File 1. TIDieR Checklist**

| 1. **Project name:** |  |
| --- | --- |
| 1. **Why is the project being done?**   *Project rationale, theory, or goal of the intervention* |  |
| 1. **What materials will be used in the intervention?** |  |
| 1. **What are the intervention activities?** |  |
| 1. **Where will the intervention be delivered?** |  |
| 1. **Who will provide the intervention** |  |
| 1. **How will the intervention be delivered?** |  |
| 1. **How often will the intervention be delivered?** |  |
| 1. **IF the intervention will be tailored to any specific sites/audience, describe process:** |  |
| *Document as intervention takes place (for reporting):*   1. *Describe intervention modifications through the course of study:* |  |
| 1. *Did intervention proceed as planned? If not, describe what, why, when and how intervention was modified:* |  |
| 1. *Describe fidelity assessment:* |  |
