# Supplementary material for: Implementation evaluation of a collective impact initiative to promote adolescent health in Oklahoma County, USA
Source: BMC Public Health. 2022 Jan 10;22:57. doi: 10.1186/s12889-021-12482-1 (PMC8743353; doi:10.1186/s12889-021-12482-1)
Supplement: Supplementary file 2 — Additional file 2. Adapted Performance Measures for the Central Oklahoma Teen Pregnancy Prevention Collaboration. [file 12889_2021_12482_MOESM2_ESM.docx]

**Additional File 2. Adapted Performance Measures for the Central Oklahoma Teen Pregnancy Prevention Collaboration**

| **Domain** | **Revised Performance Measure** |
| --- | --- |
| Dosage | Number of organizations that attended at least 75% of meetings |
|  | Average organization meeting attendance |
|  | Average length of collaboration and working group meetings |
| Engagement and Training | Number of organizations planned to be engaged |
|  | Number of organizations engaged during the reporting period |
|  | Number of organizations trained through engagement with the collaboration |
|  | Number of trainings conducted by and for the collaboration |
| Fidelity and Quality | Overall quality of programming (observation form) |
|  | Number of meetings and working groups observed |
|  | Number of meetings and working groups implemented per 6 month period |
|  | Number of meetings planned for each working group |
|  | Number of meetings conducted for each working group |
|  | Items implemented through working groups during the reporting period |
