# Supplementary material for: Implementation evaluation of a collective impact initiative to promote adolescent health in Oklahoma County, USA
Source: BMC Public Health. 2022 Jan 10;22:57. doi: 10.1186/s12889-021-12482-1 (PMC8743353; doi:10.1186/s12889-021-12482-1)
Supplement: Supplementary file 3 — Additional file 3. Adapted Meeting Effectiveness Assessment. [file 12889_2021_12482_MOESM3_ESM.docx]

**Additional File 3. Adapted Meeting Effectiveness Assessment**

| **Question** | **Type of Response** | **Meeting Effectiveness Domains** | **Collective Impact Core Conditions** |
| --- | --- | --- | --- |
| **How clear were the goals from the agenda of this meeting to you?** | Likert Scale | Productivity | Common Agenda |
| **What was the general level of participation in this meeting?** | Likert Scale | Participation | Continuous Communication,  Mutually Aligned Activities |
| **Who chaired the meeting?** | Individual Selection | Leadership | Backbone Organization Support |
| **What was leadership like in this meeting?** | Likert Scale | Leadership | Backbone Organization Support |
| **What was the quality of the decision-making at this meeting?** | Likert Scale | Decision-Making | Common Agenda |
| **What was the cohesiveness among the members at this meeting?** | Likert Scale | Productivity, Conflict Resolution | Common Agenda, Mutually Aligned Activities |
| **Was there conflict present at this meeting?** | Individual Selection | Conflict Resolution | Continuous Communication |
| **If there was conflict present, was the conflict resolved?**  **If the conflict was not resolved, please check why.** | Individual Selection | Conflict Resolution | Continuous Communication |
| **How well was the meeting organized?** | Likert Scale | Leadership,  Productivity | Backbone Organization Support |
| **How productive was the meeting?** | Likert Scale | Productivity | Common Agenda, Continuous Communication, Mutually Aligned Activities |
| **What could have been done to make this meeting more effective?** | Open-Ended | Productivity | Continuous Communication |
| **What data or information was shared regarding individual organization initiatives?** | Open-Ended | - | Shared Measurement |
| **Please provide any additional comments you would like to make about this meeting.** | Open-Ended | - | - |
