# Supplementary material for: Implementation evaluation of a collective impact initiative to promote adolescent health in Oklahoma County, USA
Source: BMC Public Health. 2022 Jan 10;22:57. doi: 10.1186/s12889-021-12482-1 (PMC8743353; doi:10.1186/s12889-021-12482-1)
Supplement: Supplementary file 4 — Additional file 4. Semi-Structured Interview Protocol. [file 12889_2021_12482_MOESM4_ESM.docx]

**Additional File 4. Semi-Structured Interview Protocol**

| **Question** | **CFIR**  **Domain** | **Community Capacity Construct** | **Collective Impact Core Conditions** |
| --- | --- | --- | --- |
| To start, please describe the Central Oklahoma Teen Pregnancy Prevention Collaboration in your own words. | - | - | Common Agenda |
| What are your main responsibilities for the Collaboration?  How are you involved in the OPA Tier 2 grant?  How does your role support the goal of the Collaboration? | Characteristics of Individuals | Skills | Mutually Aligned Activities |
| We want to get some historical context for the Collaboration. How did this collaboration emerge? | Planning | Community Power |  |
| Were shared goals and objectives developed to jointly plan the Central Oklahoma Teen Pregnancy Prevention Collaboration directions? | Planning | Interorganizational  Networks | Shared Agenda |
| Who provides the leadership for the Collaboration?  Who provides the administrative support for the Collaboration?  In what ways does this infrastructure provide the leadership, support, and guidance partners need to do their work as planned? | Inner Setting | Leadership | Backbone Organization Support |
| Do you feel like the OPA Tier 2 Grant fits well within the scope and mission of the larger Collaboration? | - | - | Shared Agenda |
| What other projects are currently priorities for the Collaboration? | - | - | Shared Agenda |
| How well do you believe the Collaboration is functioning at this time? | Inner Setting | Interorganizational Networks | Continuous Communication |
| To what extent does the collaboration have a common understanding of the problem and a joint approach to solving it? | Intervention Characteristics | Critical Reflection | Shared Agenda |
| Now we want to find out a bit more about the fit of the collaborative in Central Oklahoma. Do people within Central Oklahoma know about the Collaboration? | Outer Setting | Sense of Community | - |
| How is Thrive, the backbone, specifically viewed by the Central Oklahoma community and organizations? | Outer Setting | Sense of Community | - |
| In what ways does the Collaboration’s leadership engage community members and other key stakeholders to ensure a broad-based support for the initiative? | Inner Setting | Interorganizational Networks | Backbone Organization Support |
| Has the OPA Tier 2 funding contributed to other projects within or outside of the Collaboration? | Outer Setting | Interorganizational Networks | Backbone Organization Support |
| Has the OPA Tier 2 project benefitted from other local projects that are externally funded? | Inner Setting | Interorganizational Networks | Backbone Organization Support |
| Do you think other organizations/different sectors would be interested in participating in future initiatives of the Collaboration? | Outer Setting | - | - |
| Have members of the Collaboration had an opportunity to reflect upon lessons learned and/or opportunities to improve the collaboration?  Have members had an opportunity to reflect on the OPA Tier 2 project? | Process | Critical Reflections | Continuous Communication |
| What do you think is the biggest success of the Collaboration?  Biggest success of the OPA Tier 2 project? | Process | Critical Reflections | Continuous Communication |
| What do you think is the biggest challenge of the Collaboration?  Biggest challenge of the OPA Tier 2 project? | Process | Critical Reflections | Continuous Communication |
| Is there anything else that you would like to tell us related to the Central Oklahoma Teen Pregnancy Collaboration program planning? | - | - | - |
